# Incontinence of Urine in Children, Treated with Atropia

**Published:** 1895-02

**Authors:** T. P. Satterwhite

**Affiliations:** Louisville, Ky.


### INCONTINENCE OF URINE IN CHILDREN, TREATED WITH ATROPIA.

BY T. P. SATTERWHITE, M.D.,
LOUISVILLE, KY.

Incontinence is a malady more frequent in boys than girls. It may come on at any period of childhood life, and often continues until puberty if not relieved. Many persons having the control of such children resort to punishment for a cure; the ignorant, and I regret to say more intelligent people, are often cruel to the little subjects.

It should be the duty of all medical men, whenever they are consulted on this subject, to take especial pains to explain that the trouble is beyond control of the child, that punishment is not only fruitless but absolutely cruel, and not infrequently assists in perpetuating the habit.

The causes of incontinence are numerous, requiring careful analysis to determine the source. Simple enuresis is purely a nervous trouble. That occurring not only at night but during the day may be caused from some congenital malformation or from reflex trouble. The nocturnal incontinence is far more frequent and less serious than when it occurs both day and night.

Some of the causes that produce this affliction are atrophy of the bladder, overflow from vesical paralysis, phimosis or adherent prepuce, leucorrheal discharge in little girls, calculus in the urethra or bladder, an impacted fecal mass in the rectum, the round or thread worm, or any rectal or intestinal irritation; in fact, irritation of the remotest part of the body may cause incontinence. Cystitis will cause loss of control of the bladder, and when severe enough there will be no difficulty in determining the cause in this instance. Indigestion is a prolific cause; excessive acidity of the urine is frequently an exciting cause. Improper feeding at night or late in the evening may produce gastric disturbance, which, by its reflex irritation, causes wetting of the bed. When the cause cannot be discovered our treatment will of course have to be experimental. The general health in all cases should receive our first attention. The disease cannot be cured as long as the digestive apparatus is disturbed. Poor health is a potent factor in maintaining enuresis. The child should have a liberal amount of strictly wholesome food ; a very light supper and but little fluid during the evening. If there is hyperacidity of the urine that must be corrected. We must see that the patient is kept in the best possible general health and remove any reflex trouble that may be discovered.

The treatment by belladonna has been a practice of such long standing that probably every member of this society can remember when he commenced the practice of medicine this remedy was recommended and used. Various results have been obtained from its use, but its long continued employment in this trouble shows its usefulness notwithstanding the varied experiences of practitioners. This varied experience is due, in my opinion, to two causes: first, that the incontinence was not due to a neurosis simply but to a reflex trouble which required surgical interference ; second, that the remedy was not pushed to its full physiological effect and continued long enough.

When the smallest physiological dose of atropia is administered the only symptom is dryness of the throat and mouth, possibly some disordered vision. When a larger amount is given this dryness becomes more intense and is associated with redness of the fauces, dilated pupils, disordered vision, and possibly diplopia. Often from the first, certainly after a short time, in all cases, the heart's beat becomes rapid, and after a large dose of the alkaloid exceedingly rapid, often accompanied by a peculiar red flush on the face and neck, which may spread over the whole body ; in very severe exhibitions of the rash, desquamation of the skin sometimes follows. Intelligence may remain perfect, but there is generally some lightness of head, giddiness, and confusion of thought, as well as a staggering gait. Even with doses that are medicinal there are spectral illusions. Drowsiness is not a general or at all characteristic symptom. When a decidedly poisonous dose of belladonna or its alkaloid has been taken, all these symptoms are intensified; sometimes the patients are exceedingly violent, and convulsions may appear, followed by stupor and paralysis. Lividity of the face showing imperfect aëration of the blood is not seen in atropia poisoning except in a stage of most imminent peril. Death is preceded by marked heart and respiratory failure.

Upon the muscular structure of the heart itself atropia acts as a depressant, but it would have to be taken in very large amounts to be apparent; on the other hand, atropia acts, it is claimed, on the cardiac nerve centers as a stimulant, and unless taken in very large amounts it does not destroy excitability of these nerves.

Evidence is directly in favor of the fact that atropia in small doses contracts the capillaries, and only when poisonous doses are given do they become dilated. Atropia acts on the peripheral filaments of the nerves; it is mainly eliminated by the kidneys, and its local action on the nerve filaments of the bladder, I have no doubt, is one of the modes of relief for incontinence, when the interior of the bladder is the seat of the trouble. We are all familiar with the local action of belladonna to relieve pain as in myalgia, lumbago, pleurodenia, etc.

In the last eighteen months I have treated five or six cases of nightly incontinence, all of which responded satisfactorily to the atropia treatment when administered to its full physiological effect. In all my cases I used the metric granules, manufactured in Philadelphia, commencing with the one five-hundreth of a grain three times per day, increasing the dose gradually for the first few days. After that the increase was more rapid until there were some decided symptoms of distress; even then the dose was continued cautiously, and the toxic symptoms would often disappear without decreasing the amount. In only three cases did I have to gradually decrease the dose that was being administered. It is also necessary in the treatment to require the child brought under your daily observation to consider the propriety of increasing, maintaining, or decreasing the medicine. I find we cannot intrust to the parent the dose that is to be administered, and it is not improbable the mental effect of visiting the doctor every day is beneficial.

In one case a boy, age nine years, had wetted the bed every night from birth, and seldom less than twice a night. The first dose administered was one five-hundreth of a grain; it produced such nausea, with vomiting and general redness of the surface, that his parents were alarmed. I decreased the dose slightly for several days; the child that week soiled the bed only three times. The dose was then gradually increased daily with the result, at the end of the second week, of a slightly improved record. The dose at the end of the third week had gotten to one one-hundreth of a grain three times per day, with the result of additional improvement. The atropia was increased to one-fiftieth of a grain before the child was cured. Singularly to state even at this dose, although the pupils were fully dilated, he never complained of his vision or any other unpleasant symptom. Nor did any of the children I have treated, though they played out in the sunlight.

The second child was eight years of age, a robust, healthy looking boy, who had been soiling the bed for five years. He was relieved very promptly and I withdrew the medicine abruptly. The incontinence returned in a few nights and I had to recommence the treatment. The medicine was then gradually withdrawn and the cure was completed.

